# *N*′-[(1*E*)-(4-Fluoro­phen­yl)methyl­idene]thio­phene-2-carbohydrazide

**DOI:** 10.1107/S1600536811056121

**Published:** 2012-01-11

**Authors:** Amer M. Alanazi, Siham Lahsasni, Ali A. El-Emam, Seik Weng Ng

**Affiliations:** a Department of Pharmaceutical Chemistry, College of Pharmacy, King Saud University, Riyadh 11451, Saudi Arabia; b Department of Chemistry, University of Malaya, 50603 Kuala Lumpur, Malaysia; c Chemistry Department, Faculty of Science, King Abdulaziz University, PO Box 80203 Jeddah, Saudi Arabia

## Abstract

In the title compound, C_12_H_9_FN_2_OS, the thienyl ring is disordered over two positions, with the S atom of the major component [occupancy = 87.08 (16)°] oriented towards the *ortho*-H atom of the benzene ring. The mol­ecule is nearly planar, the dihedral angle between the thio­phene and benzene rings being 13.0 (2)° in the major component. The azomethine C=N double bond in the mol­ecule is of an *E* configuration. In the crystal, mol­ecules are linked by pairs of N—H⋯O hydrogen bonds, forming inversion dimers.

## Related literature

For the 4-chloro and 4-bromo derivatives, see: Jiang (2010*a* ▶,*b* ▶).
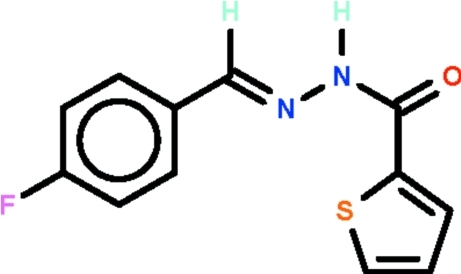


## Experimental

### 

#### Crystal data

C_12_H_9_FN_2_OS
*M* *_r_* = 248.27
Monoclinic, 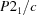

*a* = 13.3076 (11) Å
*b* = 5.6015 (4) Å
*c* = 15.3062 (12) Å
β = 104.166 (9)°
*V* = 1106.27 (15) Å^3^
*Z* = 4
Mo *K*α radiation
μ = 0.29 mm^−1^
*T* = 100 K
0.35 × 0.15 × 0.05 mm

#### Data collection

Agilent SuperNova Dual diffractometer with an Atlas detector
Absorption correction: multi-scan (*CrysAlis PRO*; Agilent, 2010 ▶) *T*_min_ = 0.906, *T*_max_ = 0.986
4609 measured reflections
2532 independent reflections
1917 reflections with *I* > 2σ(*I*)
*R*_int_ = 0.037

#### Refinement

*R*[*F* ^2^ > 2σ(*F* ^2^)] = 0.043
*wR*(*F* ^2^) = 0.108
*S* = 1.06
2532 reflections
171 parameters
5 restraints
H atoms treated by a mixture of independent and constrained refinement
Δρ_max_ = 0.30 e Å^−3^
Δρ_min_ = −0.28 e Å^−3^

### 

Data collection: *CrysAlis PRO* (Agilent, 2010 ▶); cell refinement: *CrysAlis PRO*; data reduction: *CrysAlis PRO*; program(s) used to solve structure: *SHELXS97* (Sheldrick, 2008 ▶); program(s) used to refine structure: *SHELXL97* (Sheldrick, 2008 ▶); molecular graphics: *X-SEED* (Barbour, 2001 ▶); software used to prepare material for publication: *publCIF* (Westrip, 2010 ▶).

## Supplementary Material

Crystal structure: contains datablock(s) global, I. DOI: 10.1107/S1600536811056121/xu5417sup1.cif

Structure factors: contains datablock(s) I. DOI: 10.1107/S1600536811056121/xu5417Isup2.hkl

Additional supplementary materials:  crystallographic information; 3D view; checkCIF report

## Figures and Tables

**Table 1:** Hydrogen-bond geometry (Å, °)

*D*—H⋯*A*	*D*—H	H⋯*A*	*D*⋯*A*	*D*—H⋯*A*
N1—H1⋯O1^i^	0.90 (2)	1.99 (3)	2.893 (2)	176 (2)

Symmetry code: (i) 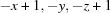.

**Fig. 1.**
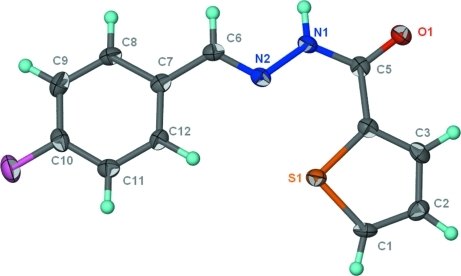
Thermal ellipsoid plot (Barbour, 2001) of C12H9FN2OS at the 70% probability level; hydrogen atoms are drawn as spheres of arbitrary radius. The disorder is not shown.

## supplementary crystallographic information

### Comment

2-Thienoylhydrazide forms a large number of Schiff base derivatives with substituted benzaldehydes; among those whose crystal structures have been reported are the 4-chloro and 4-bromo derivatives (Jiang, 2010*a*, 2010*b*). However, the 4-fluoro analog (Scheme I) is disordered in respect of the thienyl ring (Fig. 1). The azomethine double-bond in the approximately planar C_12_H_9_FN_2_OS molecule is of an *E* configuration. The thienyl ring is disordered over two positions, with the S atom of the major component (87.1 (2) %) oriented towards the *ortho*-H atom of the benzene ring. Two molecules are linked across a center-of-inversion by an N–H···O hydrogen bond to generate a dimer (Table 1).

### Experimental

2-Thienoylhydrazide (1.42 g, 0.01 mol) and 4-fluorobenzaldehyde (1.24 g, 0.01 mol) dissolved in ethanol (8 ml) was heated for 1 h. The product was collected and recystallized from ethanol to yield the Schiff base in 90% yield, m.p. 447–448 K.

### Refinement

Carbon-bound H-atoms were placed in calculated positions [C–H 0.95 Å, *U*_iso_(H) 1.2*U*_eq_(C)] and were included in the refinement in the riding model approximation.

The amino H-atom was located in a difference Fourier map, and was refined freely refined.

The thienyl ring is disordered over two positions in respect of four of the five atoms, with major component being 87.1 (2) %. Pairs of C–C and C–S bond distances were restrained to within 0.0 Å of each other. The temperature factors of C3' was set to those of S1 (as were these pairs: C2' to C1, C1' to C2 and S1' to C3).

### Crystal data

**Table:**

C_12_H_9_FN_2_OS	*F*(000) = 512
*M**_r_* = 248.27	*D*_x_ = 1.491 Mg m^−^^3^
Monoclinic, *P*2_1_/*c*	Mo *K*α radiation, λ = 0.71073 Å
Hall symbol: -P 2ybc	Cell parameters from 1715 reflections
*a* = 13.3076 (11) Å	θ = 2.7–27.5°
*b* = 5.6015 (4) Å	µ = 0.29 mm^−^^1^
*c* = 15.3062 (12) Å	*T* = 100 K
β = 104.166 (9)°	Prism, colorless
*V* = 1106.27 (15) Å^3^	0.35 × 0.15 × 0.05 mm
*Z* = 4	

### Data collection

**Table:**

Agilent SuperNova Dual diffractometer with an Atlas detector	2532 independent reflections
Radiation source: SuperNova (Mo) X-ray Source	1917 reflections with *I* > 2σ(*I*)
Mirror	*R*_int_ = 0.037
Detector resolution: 10.4041 pixels mm^-1^	θ_max_ = 27.6°, θ_min_ = 2.7°
ω scan	*h* = −13→17
Absorption correction: multi-scan (CrysAlis PRO; Agilent, 2010)	*k* = −7→4
*T*_min_ = 0.906, *T*_max_ = 0.986	*l* = −19→11
4609 measured reflections	

### Refinement

**Table:**

Refinement on *F*^2^	Primary atom site location: structure-invariant direct methods
Least-squares matrix: full	Secondary atom site location: difference Fourier map
*R*[*F*^2^ > 2σ(*F*^2^)] = 0.043	Hydrogen site location: inferred from neighbouring sites
*wR*(*F*^2^) = 0.108	H atoms treated by a mixture of independent and constrained refinement
*S* = 1.06	*w* = 1/[σ^2^(*F*_o_^2^) + (0.0425*P*)^2^ + 0.113*P*] where *P* = (*F*_o_^2^ + 2*F*_c_^2^)/3
2532 reflections	(Δ/σ)_max_ = 0.001
171 parameters	Δρ_max_ = 0.30 e Å^−^^3^
5 restraints	Δρ_min_ = −0.28 e Å^−^^3^

### Fractional atomic coordinates and isotropic or equivalent isotropic displacement parameters (Å^2^)

**Table:**

	*x*	*y*	*z*	*U*_iso_*/*U*_eq_	Occ. (<1)
S1	0.72252 (5)	0.67192 (12)	0.49050 (4)	0.01785 (19)	0.8708 (16)
S1'	0.5631 (6)	0.5848 (19)	0.3160 (5)	0.0218 (7)	0.1292 (16)
F1	1.06801 (9)	0.4889 (2)	0.93514 (8)	0.0291 (3)	
O1	0.49118 (10)	0.2092 (2)	0.40405 (9)	0.0203 (3)	
N1	0.60280 (12)	0.2114 (3)	0.54014 (11)	0.0181 (4)	
N2	0.68783 (12)	0.3050 (3)	0.59944 (11)	0.0164 (4)	
C1	0.7215 (3)	0.8617 (10)	0.4025 (4)	0.0181 (7)	0.8708 (16)
H1A	0.7675	0.9928	0.4055	0.022*	0.8708 (16)
C2	0.6467 (8)	0.8018 (16)	0.3276 (5)	0.0203 (9)	0.8708 (16)
H2	0.6347	0.8863	0.2722	0.024*	0.8708 (16)
C3	0.5900 (3)	0.6034 (9)	0.3415 (3)	0.0218 (7)	0.8708 (16)
H3	0.5354	0.5381	0.2958	0.026*	0.8708 (16)
C1'	0.642 (6)	0.827 (12)	0.318 (4)	0.0203 (9)	0.13
H1'	0.6442	0.9298	0.2691	0.024*	0.1292 (16)
C2'	0.702 (3)	0.842 (8)	0.404 (3)	0.0181 (7)	0.13
H2'	0.7500	0.9690	0.4205	0.022*	0.1292 (16)
C3'	0.6923 (14)	0.671 (3)	0.4673 (13)	0.01785 (19)	0.13
H3'	0.7302	0.6679	0.5286	0.021*	0.1292 (16)
C4	0.61940 (14)	0.5099 (3)	0.42645 (12)	0.0166 (4)	
C5	0.56736 (14)	0.3024 (3)	0.45575 (13)	0.0165 (4)	
C6	0.71912 (15)	0.1929 (3)	0.67428 (13)	0.0169 (4)	
H6	0.6836	0.0534	0.6854	0.020*	
C7	0.80912 (14)	0.2777 (3)	0.74281 (12)	0.0162 (4)	
C8	0.84780 (15)	0.1384 (3)	0.81905 (13)	0.0188 (4)	
H8	0.8139	−0.0068	0.8264	0.023*	
C9	0.93487 (16)	0.2074 (3)	0.88442 (13)	0.0227 (5)	
H9	0.9613	0.1112	0.9360	0.027*	
C10	0.98170 (15)	0.4193 (4)	0.87216 (13)	0.0199 (4)	
C11	0.94486 (15)	0.5653 (3)	0.79870 (13)	0.0181 (4)	
H11	0.9783	0.7121	0.7926	0.022*	
C12	0.85802 (14)	0.4930 (3)	0.73393 (12)	0.0166 (4)	
H12	0.8316	0.5913	0.6829	0.020*	
H1	0.5728 (17)	0.078 (4)	0.5544 (15)	0.035 (7)*	

### Atomic displacement parameters (Å^2^)

**Table:**

	*U*^11^	*U*^22^	*U*^33^	*U*^12^	*U*^13^	*U*^23^
S1	0.0196 (4)	0.0175 (3)	0.0169 (4)	−0.0051 (3)	0.0053 (2)	−0.0012 (2)
S1'	0.010 (2)	0.0290 (13)	0.025 (2)	−0.0022 (16)	0.0011 (14)	0.0011 (18)
F1	0.0230 (6)	0.0378 (7)	0.0222 (7)	−0.0021 (6)	−0.0027 (5)	−0.0023 (6)
O1	0.0173 (7)	0.0236 (7)	0.0197 (7)	−0.0040 (6)	0.0037 (6)	−0.0009 (6)
N1	0.0172 (8)	0.0194 (9)	0.0174 (9)	−0.0056 (7)	0.0035 (7)	−0.0015 (7)
N2	0.0146 (8)	0.0165 (8)	0.0185 (9)	−0.0005 (7)	0.0048 (7)	−0.0025 (6)
C1	0.015 (2)	0.0156 (15)	0.0255 (11)	−0.0038 (15)	0.0096 (15)	0.0012 (9)
C2	0.0175 (15)	0.022 (3)	0.023 (2)	0.004 (2)	0.0069 (15)	0.0076 (13)
C3	0.010 (2)	0.0290 (13)	0.025 (2)	−0.0022 (16)	0.0011 (14)	0.0011 (18)
C1'	0.0175 (15)	0.022 (3)	0.023 (2)	0.004 (2)	0.0069 (15)	0.0076 (13)
C2'	0.015 (2)	0.0156 (15)	0.0255 (11)	−0.0038 (15)	0.0096 (15)	0.0012 (9)
C3'	0.0196 (4)	0.0175 (3)	0.0169 (4)	−0.0051 (3)	0.0053 (2)	−0.0012 (2)
C4	0.0148 (9)	0.0168 (9)	0.0192 (10)	0.0011 (8)	0.0058 (8)	−0.0020 (8)
C5	0.0151 (9)	0.0186 (10)	0.0169 (10)	0.0012 (8)	0.0062 (8)	−0.0030 (8)
C6	0.0176 (10)	0.0153 (9)	0.0195 (10)	−0.0010 (8)	0.0077 (8)	0.0006 (8)
C7	0.0181 (10)	0.0168 (9)	0.0159 (9)	0.0017 (8)	0.0084 (8)	−0.0005 (7)
C8	0.0225 (10)	0.0151 (9)	0.0204 (10)	−0.0016 (8)	0.0082 (9)	0.0013 (8)
C9	0.0280 (11)	0.0218 (10)	0.0174 (10)	0.0058 (9)	0.0036 (9)	0.0040 (8)
C10	0.0161 (9)	0.0268 (11)	0.0162 (10)	0.0004 (9)	0.0030 (8)	−0.0043 (8)
C11	0.0200 (10)	0.0166 (10)	0.0195 (10)	−0.0013 (8)	0.0083 (9)	−0.0014 (8)
C12	0.0177 (10)	0.0177 (9)	0.0151 (9)	0.0034 (8)	0.0055 (8)	0.0013 (8)

### Geometric parameters (Å, °)

**Table:**

S1—C1	1.714 (4)	C2'—C3'	1.389 (11)
S1—C4	1.7346 (19)	C2'—H2'	0.9500
S1'—C1'	1.713 (11)	C3'—C4	1.361 (10)
S1'—C4	1.725 (6)	C3'—H3'	0.9500
F1—C10	1.363 (2)	C4—C5	1.478 (3)
O1—C5	1.238 (2)	C6—C7	1.465 (3)
N1—C5	1.361 (2)	C6—H6	0.9500
N1—N2	1.370 (2)	C7—C12	1.392 (3)
N1—H1	0.90 (2)	C7—C8	1.394 (3)
N2—C6	1.283 (2)	C8—C9	1.388 (3)
C1—C2	1.363 (3)	C8—H8	0.9500
C1—H1A	0.9500	C9—C10	1.374 (3)
C2—C3	1.388 (5)	C9—H9	0.9500
C2—H2	0.9500	C10—C11	1.380 (3)
C3—C4	1.367 (4)	C11—C12	1.386 (3)
C3—H3	0.9500	C11—H11	0.9500
C1'—C2'	1.362 (10)	C12—H12	0.9500
C1'—H1'	0.9500		
			
C1—S1—C4	91.55 (18)	C5—C4—S1'	111.4 (3)
C1'—S1'—C4	93.3 (15)	C3—C4—S1	109.9 (2)
C5—N1—N2	121.58 (17)	C5—C4—S1	127.20 (14)
C5—N1—H1	118.1 (14)	O1—C5—N1	119.21 (18)
N2—N1—H1	120.0 (14)	O1—C5—C4	120.57 (17)
C6—N2—N1	116.04 (16)	N1—C5—C4	120.22 (17)
C2—C1—S1	111.8 (3)	N2—C6—C7	120.68 (17)
C2—C1—H1A	124.1	N2—C6—H6	119.7
S1—C1—H1A	124.1	C7—C6—H6	119.7
C1—C2—C3	112.4 (4)	C12—C7—C8	118.76 (18)
C1—C2—H2	123.8	C12—C7—C6	122.06 (17)
C3—C2—H2	123.8	C8—C7—C6	119.18 (17)
C4—C3—C2	114.3 (3)	C9—C8—C7	121.31 (18)
C4—C3—H3	122.8	C9—C8—H8	119.3
C2—C3—H3	122.8	C7—C8—H8	119.3
C2'—C1'—S1'	106 (3)	C10—C9—C8	117.96 (18)
C2'—C1'—H1'	127.0	C10—C9—H9	121.0
S1'—C1'—H1'	127.0	C8—C9—H9	121.0
C1'—C2'—C3'	120 (4)	F1—C10—C9	118.83 (17)
C1'—C2'—H2'	120.0	F1—C10—C11	118.46 (17)
C3'—C2'—H2'	120.0	C9—C10—C11	122.72 (18)
C4—C3'—C2'	108 (2)	C10—C11—C12	118.51 (18)
C4—C3'—H3'	125.9	C10—C11—H11	120.7
C2'—C3'—H3'	125.9	C12—C11—H11	120.7
C3'—C4—C5	135.8 (9)	C11—C12—C7	120.73 (17)
C3—C4—C5	122.9 (2)	C11—C12—H12	119.6
C3'—C4—S1'	112.3 (9)	C7—C12—H12	119.6
			
C5—N1—N2—C6	−174.53 (16)	N2—N1—C5—O1	178.84 (15)
C4—S1—C1—C2	−0.7 (8)	N2—N1—C5—C4	−1.2 (3)
S1—C1—C2—C3	0.2 (13)	C3'—C4—C5—O1	168.9 (14)
C1—C2—C3—C4	0.5 (13)	C3—C4—C5—O1	−2.7 (4)
C4—S1'—C1'—C2'	−3(7)	S1'—C4—C5—O1	−2.1 (4)
S1'—C1'—C2'—C3'	2(9)	S1—C4—C5—O1	176.76 (14)
C1'—C2'—C3'—C4	0(7)	C3'—C4—C5—N1	−11.1 (14)
C2'—C3'—C4—C3	−1(3)	C3—C4—C5—N1	177.3 (3)
C2'—C3'—C4—C5	−174 (2)	S1'—C4—C5—N1	177.9 (4)
C2'—C3'—C4—S1'	−3(3)	S1—C4—C5—N1	−3.2 (3)
C2'—C3'—C4—S1	149 (7)	N1—N2—C6—C7	−179.76 (15)
C2—C3—C4—C3'	4.5 (13)	N2—C6—C7—C12	6.3 (3)
C2—C3—C4—C5	178.5 (7)	N2—C6—C7—C8	−173.20 (17)
C2—C3—C4—S1'	176 (3)	C12—C7—C8—C9	−1.6 (3)
C2—C3—C4—S1	−1.0 (8)	C6—C7—C8—C9	177.91 (17)
C1'—S1'—C4—C3'	4(4)	C7—C8—C9—C10	0.5 (3)
C1'—S1'—C4—C3	−5(5)	C8—C9—C10—F1	−179.27 (16)
C1'—S1'—C4—C5	177 (4)	C8—C9—C10—C11	0.9 (3)
C1'—S1'—C4—S1	−2(4)	F1—C10—C11—C12	179.03 (15)
C1—S1—C4—C3'	−30 (5)	C9—C10—C11—C12	−1.1 (3)
C1—S1—C4—C3	1.0 (3)	C10—C11—C12—C7	0.0 (3)
C1—S1—C4—C5	−178.5 (3)	C8—C7—C12—C11	1.3 (3)
C1—S1—C4—S1'	0.3 (5)	C6—C7—C12—C11	−178.15 (17)

### Hydrogen-bond geometry (Å, °)

**Table:**

*D*—H···*A*	*D*—H	H···*A*	*D*···*A*	*D*—H···*A*
N1—H1···O1^i^	0.90 (2)	1.99 (3)	2.893 (2)	176 (2)

Symmetry codes: (i) −*x*+1, −*y*, −*z*+1.
